# Metastatic small cell neuroendocrine carcinoma of the cervix treated with the PD-1 inhibitor, nivolumab: a case report

**DOI:** 10.1186/s40661-017-0038-9

**Published:** 2017-02-02

**Authors:** Sarah E. Paraghamian, Teresa C. Longoria, Ramez N. Eskander

**Affiliations:** Department of Obstetrics and Gynecology, Division of Gynecologic Oncology, University of California Irvine Medical Center, 33 City Blvd. West #1400, Orange, CA 92868 USA

**Keywords:** Small cell neuroendocrine carcinoma, Cervical cancer, PD-1 inhibitor, Nivolumab, Immunotherapy

## Abstract

**Background:**

Nivolumab is an immune checkpoint inhibitor specific for the programmed death 1 (PD-1) receptor that has led to clinical responses in many cancer types. Identifying biomarkers predictive of response to PD-1 blockade is an area of active investigation.

**Case presentation:**

We present a patient with recurrent, metastatic, PD-L1-negative small cell neuroendocrine carcinoma of the cervix (SCNEC) who experienced a complete response to nivolumab. Though nivolumab was discontinued over 4 months ago due to treatment-related adverse events, she continues to have no evidence of disease.

**Conclusions:**

Immune checkpoint inhibitors may be active in neuroendocrine cervical cancer, with potential for dramatic responses in a modest subset of patients.

## Background

Small cell neuroendocrine carcinoma of the cervix (SCNEC) is a rare and aggressive histology. It accounts for less than 2% of cervical cancers [1]. Unlike squamous cell and adenocarcinoma, SCNEC is more likely to have lymphovascular space invasion and lymph node involvement at the time of diagnosis [1, 2]. Patients frequently present with locally advanced tumors or distant metastases, resulting in poor oncologic outcomes with a 5-year survival rate estimated at 36.8% for early stage disease and less than 10% for advanced disease [1, 2]. Given these poor outcomes, as well as a lack of prospective data to guide treatment decisions, patients with SCNEC pose a therapeutic challenge.

SCNEC is morphologically similar to small cell lung cancer (SCLC) and treatment considerations draw on studies conducted in small cell lung cancer cohorts. For early stage disease, multimodal therapy with surgery followed by adjuvant cisplatin/etoposide with or without pelvic radiation is favored [1, 2]. More recently, results from the SCLC cohort of CheckMate 032 were published, describing durable responses in a pretreated patient population with single agent nivolumab or combination nivolumab and ipilimumab (Antonia, 2016 #2913) To date, there are no studies informing treatment of progressive or recurrent SCNEC after failure of platinum-based therapy [2]. There is an urgent, unmet clinical need to develop effective treatments.

Nivolumab is an immune checkpoint inhibitor that is specific for the programmed death 1 (PD-1) receptor. PD-1 can be expressed transiently or chronically on T cells depending on the duration of antigen exposure. The interaction of PD-1 with its ligand, PD-L1 or PD-L2, results in downstream signaling that inhibits T cell cytotoxicity and cytokine release. The rationale behind blockade of the PD-1 pathway is to abrogate an immunosuppressive mechanism present in the tumor microenvironment (TME). In this report, we present the clinical experience of a woman with recurrent, metastatic, SCNEC who had a complete response to treatment with nivolumab.

## Case presentation

A 38-year-old nulligravida with no history of abnormal pap smears presented to her primary gynecologist with complaint of malodorous brown vaginal discharge. A pap smear was performed, which returned positive for adenocarcinoma and high-risk human papillomavirus (HPV). She was subsequently referred to gynecologic oncology and diagnosed with a Federation of Gynecology and Obstetrics (FIGO) stage IB2 cervical cancer. Biopsies revealed a high-grade small cell neuroendocrine carcinoma, and positron emission tomography–computed tomography (PET/CT) showed no evidence of metastatic disease. Plan was made for radical surgical excision followed by adjuvant chemotherapy and radiation.

The patient underwent radical abdominal hysterectomy, bilateral salpingo-oophorectomy and pelvic lymphadenectomy. Pathology was negative for involvement of the surgical margins, parametria or pelvic lymph nodes. The primary tumor was 4.5 by 3.3 cm in size and involved half of the cervical stroma, with lymphovascular space involvement. Her postoperative course was complicated by a pelvic abscess requiring re-exploration and washout. Following recovery, she was treated with 6 cycles of cisplatin 80 mg/m^2^ intravenously (IV) on day 1 and etoposide 100 mg/m^2^ IV on day 1, 2 and 3. Chemotherapy was well tolerated with only grade 1 nausea and fatigue.

**Fig. 1:**
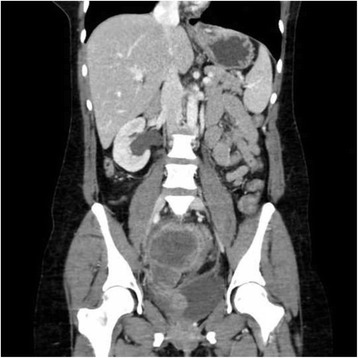
Progressive recurrent pelvic disease resulting in hydronephrosis

One month after completion of chemotherapy, surveillance pelvic exam was significant for a 1.5-cm, firm, smooth anterior vaginal wall mass. PET/CT demonstrated interval development of multiple hypermetabolic mesenteric deposits, largest measuring 21 mm with a standard uptake value (SUV) of 10.6. Shortly thereafter, she was admitted for small bowel obstruction, with imaging revealing multifocal progression of the pelvic lesions (Fig. 1). Given disease distribution, systemic chemotherapy was favored over local radiotherapy, and she received 2 cycles of paclitaxel 135 mg/m^2^ IV on day 1 and topotecan 0.75 mg/m^2^ IV on day 1, 2 and 3. Following cycle 2 of therapy, the patient was admitted for progressive pelvic pain due disease progression resulting in obstructive uropathy (Fig. 1). While hospitalized, a right percutaneous nephrostomy tube was placed, and palliative pelvic radiation therapy was initiated. The patient ultimately received a total of 37.5 Gy in 15 fractions directed towards the obstructive lesion along the right pelvic side wall. The original tumor was sent for molecular testing to help inform future therapy and approval for the off-label use of nivolumab was requested from her insurance provider.

**Fig. 2:**
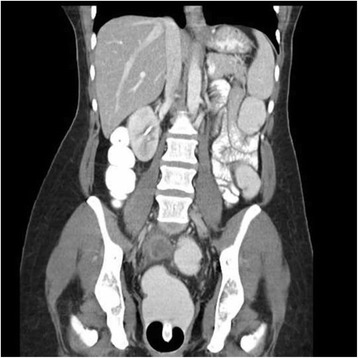
Reduction in lesion size after starting nivolumab

**Fig. 3:**
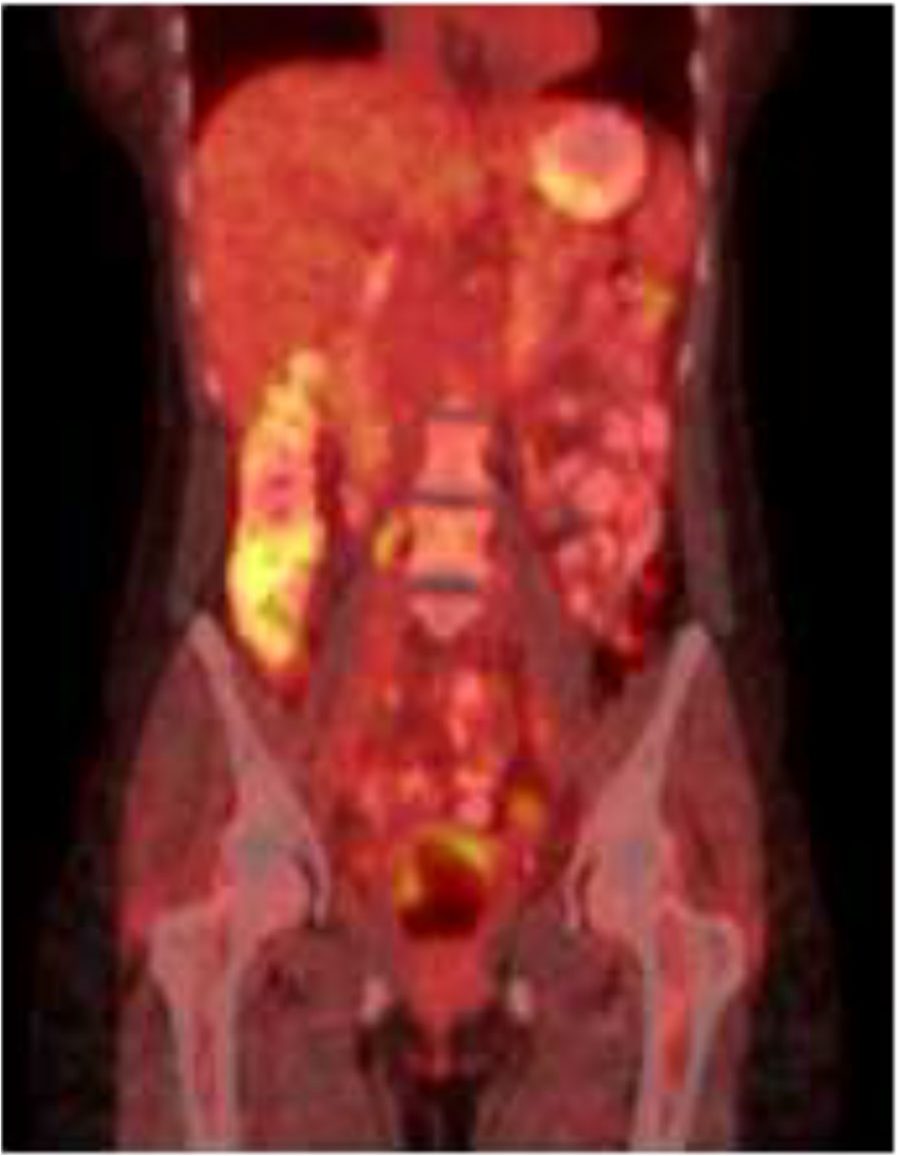
Complete resolution of all lesions after 6^th^ dose of nivolumab

Nivolumab was initiated at a dose of 3 mg/kg IV every 2 weeks prior to molecular characterization of her tumor, which showed absent PD-L1 expression. After 2 doses, radiographic imaging demonstrated a decreased in size of all target lesions (Fig. 2). Concurrently, all cell counts began to decrease. After 4 doses, the patient reported vision changes and light sensitivity. She was evaluated by ophthalmology and diagnosed with severe dry eyes and pre-glaucoma. Despite standard topical therapies, her symptoms progressed and the decision was made to discontinue treatment for persistent grade 3 ocular toxicity after the 6^th^ dose. PET/CT obtained 3 weeks after the final dose demonstrated complete resolution of all target and non-target lesions (Fig. 3). Cell counts nadired (grade 2 lymphocytopenia and thrombocytopenia, grade 3 anemia) 5 weeks after cessation of therapy and began to show significant recovery by week 7. Hematologic evaluation, inclusive of bone marrow biopsy, failed to identify an alternate source of her pancytopenia, which was attributed to nivolumab.

## Discussion

We present a patient with recurrent, metastatic, PD-L1-negative SCNEC who experienced a complete response to nivolumab therapy. Though nivolumab was discontinued over 4 months ago due to treatment-related adverse events, she continues to have no evidence of disease.

From a therapeutics standpoint, orphan diseases such as SCNEC are traditionally unable to keep pace with more common malignancies. The low likelihood of achieving sufficient patient numbers for efficacy trials discourages scientific initiatives specifically designed for rare tumor types. The typical solution to this problem has been to extrapolate treatment strategies from more frequently encountered cancers of the same cell type, such as SCLC in the case of SCNEC. This practice assumes that cell origin or morphology is the key feature to predict response to treatment. The National Cancer Institute – Molecular Analysis for Therapy Choice (NCI-MATCH) trial is evidence that this thought process is changing. In this trial, treatment with various targeted therapies is directed by genetic testing. Patients with solid tumors or lymphomas that have progressed following at least one line of standard treatment (or for which no agreed upon treatment approach exists) are assigned to a treatment arm based on the DNA sequencing results of their tumor. This strategy recognizes the heterogeneity that exists not only between different types of cancer but also between cancers of the same type.

In the search for predictive biomarkers to immune checkpoint inhibitors, the prevalence of somatic mutations has been the most promising. From the outset, mutagen-induced malignancies, namely melanoma and lung cancer, have had the greatest success in clinical trials. These cancers approach or exceed 10 somatic mutations per megabase, constituting the highest mutation frequencies of all cancers [3]. It is hypothesized that the greater the number of somatic mutations, the greater the number of neoantigens and the more immunogenic the tumor. This principle was assessed by Le and colleagues in a phase II trial designed to evaluate the clinical activity of pembrolizumab in patients with progressive metastatic carcinoma with or without mismatch-repair deficiency, which is associated with a difference of 10–100 times the number of somatic mutations [4]. They found an immune-related objective response rate of 40% and immune-related, 20-week progression-free survival rate of 78% in patients with mismatch repair–deficient colorectal cancers, compared to 0 and 11%, respectively, in patients with mismatch repair–proficient colorectal cancers. Patients with mismatch repair–deficient non-colorectal cancer had responses similar to those of patients with mismatch repair–deficient colorectal cancer.

The number of somatic mutations is unlikely to be the only determinant of tumor immunogenicity. Topalian and colleagues hypothesize that integrated oncogenic viruses are uniquely equipped to generate neoantigens that engage the immune system [5]. While point mutations or rearrangements of the tumor genome typically generate a single or limited number of T cell epitopes, the products of viral oncogenes are completely non-self and are likely to contain many more potential antigenic peptides for T cell recognition. Moreover, as drivers of tumorigenesis, products of viral oncogenes are less likely to be silenced or deleted as a mechanism of immune evasion. This theory has found support in a phase II trial of pembrolizumab in advanced Merkle-cell carcinoma [6]. Merkle-cell carcinoma, a rare but aggressive skin cancer, has been linked to 2 major causative factors, ultraviolet (UV) light and Merkle-cell polyomavirus (MCPyV), whose large T antigen is expressed in tumor cells and inactivates p53 and Rb. MCPyV-negative, UV-induced Merkel-cell carcinomas have a median of 1121 mutations per exome, which exceeds the mutational burden reported for cancers that have been most responsive to PD-1 blockade (i.e. melanoma, lung cancers, GU cancers). In contrast, MCPyV-positive Merkel-cell carcinomas, with a median of 12.5 mutations per exome, carry a mutational burden that is below cancers that have demonstrated a poor response to PD-1 blockade (i.e. prostate and pancreatic cancers). Among 25 patients assigned to pembrolizumab 2 mg per kg every 3 weeks, 44% (4 of 9 patients) of those with MCPyV-negative tumors and 62% (10 of 16 patients) of those with MCPyV-positive tumors had an objective response. In this trial, neither PD-L1 expression on tumor cells nor infiltrating immune cells was associated with clinical response to pembrolizumab.

The best correlate to Merkle-cell carcinoma in an HPV-associated cancer is head and neck squamous cell carcinoma (HNSCC). Just as in Merkle-cell carcinoma, HNSCC may be mutagen-driven (tobacco) or virus-driven, with the greater mutational burden found in the virus-negative tumors. In KEYNOTE-012, a phase Ib trial of pembrolizumab 10 mg per kg every 2 weeks in patients with PD-L1-positive recurrent or metastatic HNSCC, overall response rate (ORR) in all patients was 18% (8 of 45 patients), which consisted of 4 of 29 (14%) patients with HPV-negative tumors and 4 of 16 (25%) patients with HPV-positive tumors [7]. Similar results were found in an expansion cohort of PD-L1 positive or negative patients that received a fixed dose of pembrolizumab at a less frequent dosing schedule (200 mg IV every 3 weeks) [8]. Compared to 14% (15 of 104 patients) of patients with HPV-negative tumors, 32% (9 of 28 patients) of those with HPV-positive tumors had an objective response. Nivolumab has also performed well in recurrent or metastatic HNSCC. In CheckMate-141, a phase III trial, median overall survival (OS) was 7.5 months among patients who received nivolumab 3 mg per kg every 2 weeks compared to 5.1 months among patients who received single-agent systemic therapy [9]. There was a median OS difference of 4.7 months among patients with HPV-positive tumors (9.1 months in nivolumab group vs 4.4 months in standard-therapy group; hazard ratio for death, 0.56; 95% CI, 0.32–0.99) and a difference of 1.7 months among patients with HPV-negative tumors (7.5 months in nivolumab group versus 5.8 months in standard-therapy group; hazard ratio, 0.73; 95% CI, 0.42–1.25).

It remains to be seen whether integrated oncogenic viruses may ultimately be validated as a predictive biomarker for immune checkpoint inhibitors. While we await the results of additional trials examining checkpoint inhibition in subjects with virus-positive and virus-negative cervical cancer (NCT02488759, NCT02257528), it is important to note that complete responses and sustained responses remain rare. Of 177 patients in KEYNOTE-012 who were evaluated by central review, only 5 patients (2.8%), 4 of which where HPV-positive, demonstrated a complete response to therapy. In our patient, we hypothesize that radiotherapy may have served to sensitize or prime the immune system. Tumor-directed radiotherapy has been shown to stimulate the immune system by increasing antigen presentation and promoting a proinflammatory tumor microenvironment (TME), with well-documented changes in the cytokine milieu and expression of cell surface molecules [10]. These anti-tumor-specific immune responses may extend to distant, non-irradiated tumor sites, a phenomenon termed the abscopal effect. Using various mouse models, the combination of radiotherapy and immune checkpoint inhibitors has not only been shown to have synergistic effects on the TME [11] but also to extend survival [12, 13]. Dramatic responses to this combination have been reported in humans [14] and are being evaluated in the clinical trial setting (NCT02383212).

## Conclusions

Unlike the more common histological types of cervical cancer, SCNEC is rarely cured, even when diagnosed at an early stage. Its resistance to traditional therapies, reflected in the heterogeneity of treatment sequence described in the literature, encourages oncologists to look to novel therapies. Immunotherapy has the capacity to turn the causative agent, high-risk HPV, into a feature that may be exploited for clinical benefit. Among recurrent, chemotherapy-resistant, metastatic cervical cancer patients treated with adoptive T-cell therapy (ACT) involving a single infusion of ex vivo–expanded tumor-infiltrating T cells, HPV reactivity of the infusion product positively correlated with clinical response [15]. More research is needed to evaluate whether HPV infection is a predictive biomarker for immune checkpoint inhibitors, which have the potential for dramatic responses in a modest subset of patients.

## Abbreviations

FIGO: Federation of Gynecology and Obstetrics
HPV: Human papillomavirus
HSNCC: Head and neck squamous cell carcinoma
IV: Intravenously
MCPyV: Merkle-cell polyomavirus
ORR: Overall response rate
OS: Overall survival
PD-1: Programmed death 1
PET/CT: Positron emission tomography-computed tomography
SCLC: Small cell lung cancer
SCNEC: Small cell neuroendocrine carcinoma of the cervix
SUV: Standard uptake value
TME: Tumor microenvironment
